# Internet-Based Exercise Therapy Using Algorithms for Conservative Treatment of Anterior Knee Pain: A Pragmatic Randomized Controlled Trial

**DOI:** 10.2196/rehab.5148

**Published:** 2016-12-14

**Authors:** Tae Won Benjamin Kim, Nic Gay, Arpit Khemka, Jonathan Garino

**Affiliations:** ^1^SimpleTherapy, IncFremont, CAUnited States

**Keywords:** knee pain, conservative measures, exercise therapy, nonoperative, algorithm, home-based, physical therapy

## Abstract

**Background:**

Conservative treatment remains the first-line option, and there is significant medical evidence showing that home-based exercise therapy for the treatment of common causes of knee pain is effective. SimpleTherapy created an online platform that delivers Internet-based exercise therapy for common causes of knee pain. The system is driven by an algorithm that can process the user’s feedback to provide an adaptive exercise regimen. This triple-armed, pragmatic randomized pilot was designed to evaluate if this telerehabilitation platform is safe and effective.

**Objective:**

We hypothesized that a home-based, algorithm-driven exercise therapy program can be safe for use and even improve compliance over the standard of care, the paper handout.

**Methods:**

After an independent internal review board review and approval, the website trial.simpletherapy.com was opened. Once the trial was open for enrollment, no changes to the functionality or user interaction features were performed until the trial had closed. User accrual to the website was done using website optimization and social media postings tied to existence of knee pain. Consent was obtained online through checkboxes with third-party signature confirmation. No fees were charged to any patient. Patients were recruited online from an open access website. Outcomes were self-assessed through questionnaires with no face-to-face clinician interaction. A triple-arm randomized controlled trial was used with arm 1 being a static handout of exercises, arm 2 being a video version of arm 1, and arm 3 being a video-based, algorithm-driven system that took patient feedback and changed the exercises based on the feedback. Patients used household items and were not supervised by a physical therapist or clinician. Patients were reminded at 48-hour intervals to complete an exercise session.

**Results:**

A total of 860 users found the trial and initiated the registration process. These 860 were randomized, and the demographic distribution shows the randomization was successful. In all, 70 users completed the 6-week regimen (8.1%): 20 users were in arm 1, 33 users in arm 2, and 17 users in arm 3. There were no adverse events reported in any of the 3 arms. All outcomes were self-assessed. No adverse events were reported during or after the trial.

**Conclusions:**

Because only 8.1% of those who enrolled completed the trial, an intent-to-treat analysis did not reach statistical significance in this pilot trial. However, the completion rates are comparable to those of previous online-only trials. Given an early phase trial, no adverse events were reported. Ongoing data collection continues and will form the basis for further data on the efficacy of this intervention.

**Trial Registration:**

Clinicaltrials.gov NCT01696162; https://clinicaltrials.gov/ct2/show/NCT01696162 (Archived by WebCite at http://www.webcitation.org/6lM8jC7Gu)

## Introduction

Knee pain is one of the most common conditions seen by orthopedic surgeons and primary care physicians with an estimated prevalence of 15% to 45% of the population. The causes of knee pain remain diverse, with the most common cause being osteoarthritis [1,2]. Conservative treatment remains the first-line option, and there is significant medical evidence showing that home-based exercise therapy for the treatment of common causes of knee pain is effective [3,4].

The use of the Internet to provide wide-reaching medical therapies is increasing. The term “telemedicine” has been employed to signal this widespread interest. Within telemedicine is a subcategory called “telerehabilitation.” The American Telemedicine Association defines telerehabilitation as “the delivery of rehabilitation services via information and communication technologies.” The type of information and communication technologies can vary widely, from videoconferencing to video delivery. In some stroke studies, videoconferencing techniques were shown to be efficacious and feasible [5,6]. However, research on the application of telerehabilitation and specifically the delivery of asynchronous instructional videos for common musculoskeletal conditions such as knee pain is lacking, and the effectiveness of the application remains unknown.

SimpleTherapy created an online platform that delivers Internet-based exercise therapy for common causes of knee pain. The system is designed as a stand-alone intervention capable of expanding access as a cost-effective option to physical therapy and can complement or replace visits to a physical therapists for certain populations. The core value of the platform is an algorithm that can process the user’s feedback to provide an adaptive exercise regimen. This triple-armed, randomized controlled pilot was designed to evaluate if this telerehabilitation platform is safe and effective. Our hypotheses were that (1) unsupervised, Web-based exercise therapy could be performed safely and would relieve anterior knee pain in a properly screened population and (2) this modality would be preferred in some ways over traditional, in-person physical therapy.

## Methods

### Recruitment

After an independent internal review board review and approval (Salus Internal Review Board Protocol #413), the website trial.simpletherapy.com was opened [7]. Once the trial was open for enrollment, no changes to the functionality or user interaction features were performed until the trial had closed. The trial was registered with ClinicalTrials.gov [NCT01696162]. User accrual to the website was done using website optimization and social media postings tied to existence of knee pain. Consent was obtained online through checkboxes with third-party signature confirmation. No fees were charged to any patient. Patients accessed the site through a computer connected to the Internet without supervision. Patients were recruited online from an open access website. Outcomes were self-assessed through questionnaires with no face-to-face clinician interaction. Patients were not required to be part of an organization or other diagnosis subset. No external funding was used for this study. The trial was funded by SimpleTherapy LLC.

### Onboarding

When potential users landed on the website, they underwent a 3-part series of evaluations to ensure qualification for participating in unsupervised exercise therapy. The user would be asked to fill out the Physical Activity Readiness Questionnaire (PAR-Q), a questionnaire recommended for use by the American College of Sports Medicine to help screen participants safe for exercise (Multimedia Appendix 1). If the participant answered all of the questions appropriately, they would move onto the second screen. The participants were asked whether a doctor or medical professional had said they were safe for exercise therapy. If the answer was yes, the name of the medical professional was recorded, and the user entered into the next phase of the system. If the answer was no, the user was interviewed over the phone by a physician during which a set of questions called the Knee Exercise Eligibility Score (KEES) was used (Multimedia Appendix 2). The questions were asked verbatim with request for further clarification of the potential user’s answer. Those participants who answered these questions correctly were then entered into the next phase of the system. Computer literacy was an assumed *de facto* eligibility criterion. In order to be eligible for participation in the trial, a patient had to answer all screening questions of the PAR-Q and KEES correctly.

Once the user was screened and deemed appropriate for safe participation, the user would register. Basic demographic information was collected including gender, age, height, and weight. Participants were asked to read and electronically sign a consent form outlining the clinical trial and all of the associated risks and benefits (Multimedia Appendix 3). A third-party website was used to obtain electronic signature verification. After consenting, the patient was allocated in a parallel design into three arms: arm 1, which provided 6 static exercises for knee pain viewable only on the computer screen, meant to mimic the handouts given to patients discharged from traditional physical therapy; arm 2, which provided the same 6 exercises offered in arm 1 in video form; and arm 3, the SimpleTherapy video-based platform, which delivered a progressive sequence of 6 exercises per visit based on user input from the prior exercise session.

Software code using a random number generator performed the randomization in a 1:1:1 ratio. This randomization code was not tampered with once the trial had been launched. Investigators were not involved in the randomization process. At the 3-month mark, the number of users within each arm was assessed to ensure proper allocation.

### User Engagement

Users were then asked to perform the exercises 3 times per week for 6 weeks. Surveys were gathered from the participants at the initiation of the program and 6 weeks after the program started (Multimedia Appendix 4). The exercises were selected by orthopedic surgeons, and patients gave feedback on each exercise after a session (consisting of 6 exercises). The feedback choices were “too easy,” “just right,” “too hard,” and “it hurt.” The next session’s exercises were selected by an algorithm that incorporated user feedback. Thus each exercise session was novel to patients with respect to their experience from the previous session. The videos were designed to contain the coaching of a physical therapist or orthopedic specialist regarding form, function, and experience of each exercise. All communication was via email or on-screen instructions and was asynchronous. Patients were reminded via email every 48 hours to perform a session. Clinicians monitored pain levels and feedback but did not directly communicate with patients except to answer email questions. Compliance was measured automatically based on log-in time and feedback completion.

Compliance and pain levels were assessed at 3, 6, and 12 weeks in all 3 groups. Compliance logs were monitored in a blinded fashion, and all pain levels were self-reported using a visual analog scale and completed online without clinician assistance or guidance. The visual analog scale was used due to its long-term clinical reproducibility and accuracy. Questionnaires were not validated prior to trial implementation. Questionnaires were designed by consensus of a team of orthopedic surgeons and physical therapists.

Patients were not blinded from their intervention. A software developer who is not an author was also not blinded to each patient’s allocation. All authors were blinded through the analysis of data using spreadsheets with compliance and pain data without labels to each column. Only when statistical significance was calculated were investigators made aware of arm allocation. No privacy breaches or technical problems occurred. An adverse event was defined as any user who reported an acute inability to perform the exercises (eg, was able to extend the knee and then was unable to due to a mechanical block). A serious adverse event was defined as a user who during the trial period was required to be seen in an emergency department or hospital for the knee pain or had surgical intervention for the knee pain.

Significant attrition of users during the study occurred. As such, intention-to-treat analysis was not conducted. Those included in the statistical analysis were those users who completed the program and provided the required outcome measure. This we deem a “completion analysis,” although this does not represent a truly randomized sample. Student *t* tests were conducted to compare mean pain and University of California Los Angeles (UCLA) activity scale scores within each arm at the initial, 3-week, and 6-week time points. A Cohen *d* was calculated to evaluate for effect size. Analysis of variance was performed to evaluate whether arm allocation was associated with reported pain scores and changes in pain score at 6 weeks. *P*<.05 was considered statistically significant.

## Results

### Randomization

**Figure 1:**
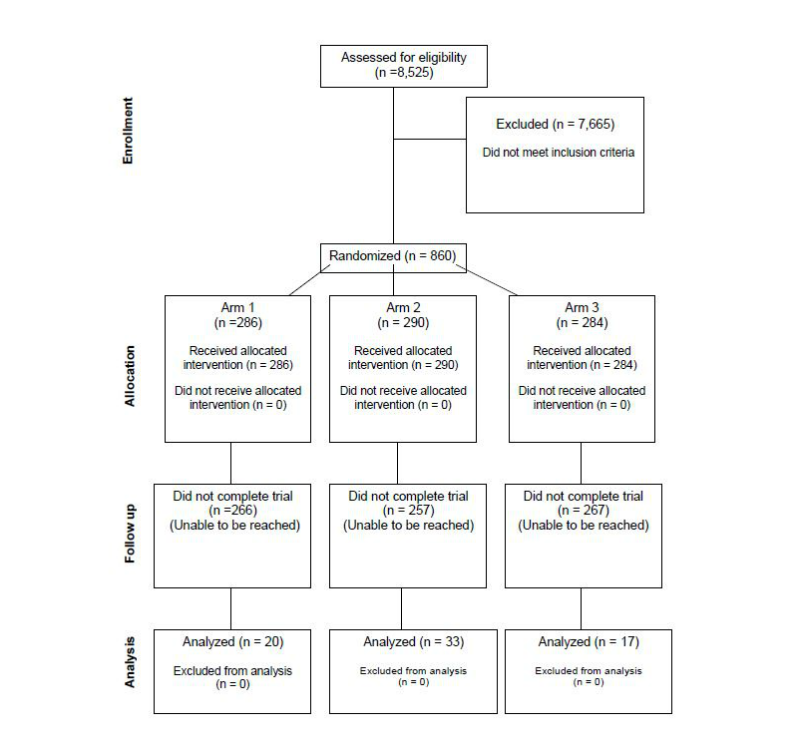
Trial onboarding and allocation flow.

**Figure 2:**
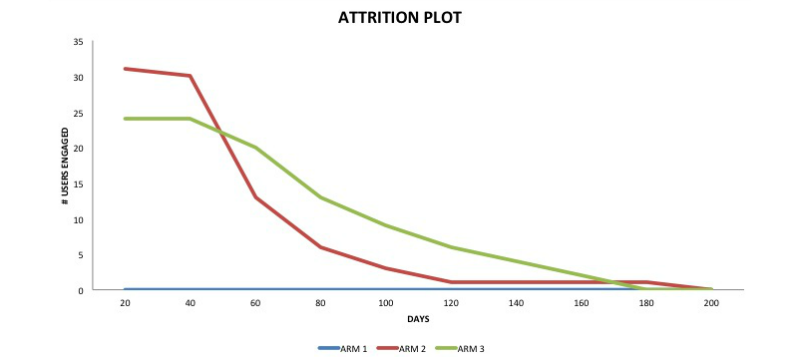
Attrition plot.

A total of 8525 individuals landed on the clinical trial website. Of these, 860 users initiated and completed the registration process. These 860 were randomized, and the demographic distribution shows the randomization was successful (Table 1). The final cohort of users who were analyzed is shown in the flow diagram in Figure 1. An attrition flow diagram indicating usage patterns is shown in Figure 2.

**Table 1:** Randomization results of users.

		Arm 1 n=286	Arm 2 n=290	Arm 3 n=284
Age (years)		52.1	51.6	51.7
**Gender**				
	Male	111	104	111
	Female	175	186	173
Weight (lb)		185.4	188.2	194.0
Body mass index (kg/m^2^)		28.0	29.2	29.1

#### Arm 1

A total of 286 users were randomized to arm 1. No users in arm 1 provided a 3-week pain or UCLA score; 20 users provided an initial and 6-week pain and UCLA activity scores. The mean initial and 6-week pain scores were 3.9 (SD 1.7, 95% CI 3.1-4.7) versus 3.7 (SD 1.8, 95% CI 2.8-4.6) (*P*=.69), respectively. Cohen *d*=0.11 *.* The mean initial and 6-week UCLA activity scores were 6.0 (SD 2.1, 95% CI 5.0-7.0) versus 6.6 (SD 2.1, 95% CI 5.6-7.6) (*P*=.23), respectively. Cohen *d*=0.29.

#### Arm 2

A total of 290 users were randomized to arm 2 with 27 users reporting an initial and 3-week pain and UCLA activity scores. The mean initial and 3-week pain scores were 4.6 (SD 1.9, 95% CI 3.9-5.3) versus 3.8 (SD 2.2, 95% CI 2.9-4.7) (*P*=.06), respectively. Cohen *d*=0.36. The mean initial and 3-week UCLA activity scores were 6.0 (SD 2.2, 95% CI 5.1-6.9) versus 6.4 (SD 1.9, 95% CI 5.6-7.2) (*P*=.27), respectively. Cohen *d*=0.19.

A total of 33 users reported an initial and 6-week pain and UCLA activity scores. The mean initial and 6-week pain scores were 4.8 (SD 1.8, 95% CI 4.2-5.4) versus 4.4 (SD 2.5, 95% CI 3.5-5.3) (*P*=.45), respectively. Cohen *d*=0.18. The mean initial and 6-week UCLA activity scores were 6.0 (SD 2.3, 95% CI 5.2-6.8) versus 6.1 (SD 2.4, 95% CI 5.3-6.9) (*P*=.8), respectively. Cohen *d*=0.04.

#### Arm 3

A total of 284 users were randomized to arm 3; 17 users reported an initial and 3-week pain and UCLA activity scores. The mean initial and 3-week pain scores were 4.4 (SD 2.2, 95% CI 3.3-5.5) versus 3.9 (SD 2.0, 95% CI 2.9-4.9) (*P*=.40), respectively. Cohen *d*=0.24. The mean initial and 3-week UCLA activity scores were 6.1 (SD 2.2, 95% CI 5.0-7.2) versus 6.8 (SD 2.5, 95% CI 5.5-8.1) (*P*=.14), respectively. Cohen *d*=0.30.

A total of 17 users reported an initial and 6-week pain and UCLA activity scores. The mean initial and 6-week pain scores were 4.5 (SD 2.1, 95% CI 3.4-5.6) versus 3.0 (SD 2.1, 95% CI 1.9-4.1) (*P*=.009), respectively. Cohen *d*=0.7. The mean initial and 6-week UCLA activity scores were 6.6 (SD 1.9, 95% CI 5.6-7.6) versus 6.6 (SD 2.0, 95% CI 5.6-7.6) (*P*>.99), respectively. Cohen *d*=0.0.

### Arm Allocation and 6-Week Pain Scores

One-way analysis of variance was conducted to compare the effects of arm allocation to reported pain score at 6 weeks as well as the change in pain score from the initially reported pain score. The mean reported pain score between groups was not significant (*P*=.11). The mean changes in pain score achieved by arms 1, 2, and 3 were −0.2 versus −0.4 versus −1.5, respectively. There was not a significant effect of arm allocation and change in pain score at the *P*<.05 level (F_2,67_=1.34, *P*=.27).

### Usability and Adverse Events

During the study, no adverse events were reported from the users. When asked whether the users enjoyed the use of this telerehabilitation platform better than in-person physical therapy, 79% (19/24) responded yes in arm 1 versus 89% (32/36) in arm 2 versus 96% (26/27) in arm 3. When asked if during the trial the user required other medical interventions such as visiting a doctor or physical therapist or receiving a knee injection, 54% (13/24) of users in arm 1 responded yes versus 22% (8/36) of users in arm 2 versus 22% (6/27) of users in arm 3 (Table 2).

Users chose the following reasons for trying the telerehabilitation platform: 8 chose “effectiveness,” 19 chose “ease of use,” 28 chose “ease of access,” 15 chose “cost,” and 17 chose “other.” Two users who chose “other” typed in their reasons: “Made sense and I could do it on my schedule” and “Doctors are too interested in invasive treatments.”

**Table 2:** Number of users who needed further medical intervention.

	None	Visited physical therapist	Visited doctor	Received injection	Med resource	%
Arm 1 (n=24)	11	3	10	0	13	54
Arm 2 (n=36)	27	0	6	2	8	22
Arm 3 (n=27)	0	0	6	0	6	22

## Discussion

### Principal Findings

Internet access and its use in health care are becoming more prevalent in the United States. The Pew Research Center recently reported that 87% of Americans use the Internet and 77% of Americans have searched online for health-related information, with the most commonly searched topics related to specific diseases or conditions and treatments. This is an increase from 62% when the survey was conducted in 2001. More than half of users aged 50 to 64 years have searched online for health information. Lastly, 28% of users went online to obtain a diagnosis. All signs point to the Internet becoming a major factor in how people access health care [8].

We hypothesized that a video-based, asynchronous Internet-only intervention could be safe and effective for patients with anterior knee pain. Safety was the number one goal of this trial, and we found that no adverse events were recorded in any of the arms. Arms 1 and 2, handouts provided to users after in-person therapy sessions and YouTube videos found on the Internet, respectively, are current standards of care accessible to the population. Comparatively, the lack of reported adverse events in the implementation of a user-feedback–based telerehabilitation algorithm (arm 3) supports the safety in providing such a service. Further, as no clinician guidance or oversight was provided, the results are generalizable to a comparable population with similar technology understanding and motivation.

We used self-reported pain scores and the UCLA activity score as a gauge of the effectiveness of the programs. The most striking finding was that after 6 weeks, users who were in arm 3 reported the lowest mean pain score compared to arm 1 and arm 2. At 3 weeks, there was no statistical difference in the mean pain score reported in arm 2 and arm 3, suggesting that the program is most effective at a minimum of 4 weeks. Furthermore, the largest reported effect size was in arm 3 at 6 weeks, supporting the idea that a user-driven telerehabilitation for anterior knee pain can be a more effective method compared to the current standards.

When looking at self-reported UCLA activity scores, there was no difference between the 3 arms, suggesting that the achieved reduction in pain did not necessarily improve activity scores. However, the UCLA activity score was designed to assess activity levels after total joint replacement. These patients have significant multicompartmental osteoarthritis and poor prejoint replacement function, allowing the UCLA activity score to capture a larger difference. Comparatively, our users’ mean starting UCLA score was 6, which correlates to users already participating in moderate activity. It may not be able to capture the subtle changes in activity that improving anterior knee pain could cause. Another activity scale may have to be employed in future studies to capture this improvement.

There was no significant difference in the changes in pain scores at 6 weeks as a function of the arm allocation. When closely looking at the absolute change, however, we find that users in arm 3 reported an average 1.5-point decrease in pain score, compared to arms 1 and 2, which each showed a less than 1-point change. This indicates a trend toward the user improving from a moderate to mild level of pain, which is clinically relevant. Further, change is unrelated to any significant increase or decrease in the UCLA activity scores, suggesting the decreasing pain level observed is directly related to the exercise regimens.

Lastly, users in arm 3, compared to arms 1 and 2, enjoyed using the program more. This is likely related to the user feeling engaged and being able to direct their own progression of exercises. Users in arm 3 showed a more than 50% reduction in the need for medical intervention such as an injection or a visit to a doctor compared to arm 1. This significant reduction in health care utilization while involved in the program is a valuable contribution to the medical community since health care costs are rising. Exercise telerehabilitation, delivered via a user feedback system, can reduce unnecessary doctor and physical therapy visits while continuing to deliver effective care.

### Limitations

Our study, however, is not without weaknesses. Only 8% of users who registered completed the 6-week system. Regularly, the difficulty of running a purely online clinical trial is evident in attrition rates. McAlindon [9] ran an online glucosamine trial for knee osteoarthritis. Patients were randomized to either a drug arm or placebo arm. A total of 1200 applicants signed up for the trial, of which 200 (16%) completed it. Although enrollment and retention were better than our current study, they spent US $950 per participant for recruitment and follow-up, which was far higher than the US $60 per person our study spent [9]. What the McAlindon study concluded was that conducting online trials was feasible and effective. The ability of our study to attract 860 users to register is comparable with another study by Formica [10]. Further, this platform was version 1.0 with few user engagement functions incorporated. We expect that with future product development, accrual and retention numbers will be significantly improved.

Secondly, our study is not sufficiently powered to evaluate efficacy in pain reduction. However, even with these small numbers, our study suggests increased effectiveness in reducing pain when users are engaged in the video user-feedback–based platform. We anticipate that future studies with greater power will demonstrate greater effectiveness. Thirdly, our data analysis was conducted as a completion analysis. Only those who provided the full data were deemed appropriate for the final analysis. This does not make this a true randomized sampling and introduces bias.

### Conclusion

In conclusion, our pilot study showed that the algorithm-driven, user-feedback–based telerehabilitation platform SimpleTherapy is safe and can be a pragmatic alternative to helping improve anterior knee pain. Since the trial, the intervention has undergone a myriad of changes to the interface; verbiage explaining the offering, reminders, and content; and the algorithm logic. Although future studies are required, the findings of this study support the continued development of this new telerehabilitation platform. We will continue to publish outcomes regarding the platform in multiple other body areas and populations. These studies are currently ongoing.

## Multimedia Appendix 1

Physical Activity Readiness Questionnaire.

## Multimedia Appendix 2

Knee Exercise Eligibility Score.

## Multimedia Appendix 3

Informed consent document.

## Multimedia Appendix 4

Questions for initial and 6-week feedback.

## Abbreviations

KEES: Knee Exercise Readiness Score
PAR-Q: Physical Activity Readiness Questionnaire
UCLA score: University of California, Los Angeles score

## References

[1] Centers for Disease Control and Prevention (1994). Prevalence of disabilities and associated health conditions: United States, 1991-1992. J Am Med Assoc.

[2] Panush RS, Lane NE (1994). Exercise and the musculoskeletal system. Baillieres Clin Rheumatol.

[3] Evcik D, Sonel B (2002). Effectiveness of a home-based exercise therapy and walking program on osteoarthritis of the knee. Rheumatol Int.

[4] Bahr R, Fossan B, Løken S, Engebretsen L (2006). Surgical treatment compared with eccentric training for patellar tendinopathy (jumper's knee). A randomized, controlled trial. J Bone Joint Surg Am.

[5] Lai JCK, Woo J, Hui E, Chan WM (2004). Telerehabilitation: a new model for community-based stroke rehabilitation. J Telemed Telecare.

[6] Reinkensmeyer DJ, Pang CT, Nessler JA, Painter CC (2002). Web-based telerehabilitation for the upper extremity after stroke. IEEE Trans Neural Syst Rehabil Eng.

[7] SimpleTherapy.

[8] (2013). Pew Research Center. Health Fact Sheet.

[9] McAlindon T, Formica M, Kabbara K, LaValley M, Lehmer M (2003). Conducting clinical trials over the Internet: feasibility study. Brit Med J.

[10] Formica M, Kabbara K, Clark R, McAlindon T (2004). Can clinical trials requiring frequent participant contact be conducted over the Internet? Results from an online randomized controlled trial evaluating a topical ointment for herpes labialis. J Med Internet Res.

