# Supplementary material for: Internet-Based Exercise Therapy Using Algorithms for Conservative Treatment of Anterior Knee Pain: A Pragmatic Randomized Controlled Trial
Source: JMIR Rehabil Assist Technol. 2016 Dec 14;3(2):e12. doi: 10.2196/rehab.5148 (PMC5454560; doi:10.2196/rehab.5148)
Supplement: Supplementary file 1 [file rehab_v3i2e12_app1.pdf]

## Multimedia Appendix 1. PAR-Q Questionnaire

### PHYSICAL ACTIVITY READINESS QUESTIONNAIRE (PAR-Q)

#### READINESS ASSESSMENT

1. Has your doctor said you have heart trouble?

   Yes No I do not know or I do not remember

2. Do you frequently suffer from pain in your chest?

   Yes No I do not know or I do not remember

3. Do you often feel faint or have spells of dizziness?

   Yes No I do not know or I do not remember

4. Has a doctor ever said your blood pressure was too high?

   Yes No I do not know or I do not remember

5. Has a doctor ever told you that you have a bone or joint problem, such as arthritis, that has been aggravated by exercise or might be made worse with exercise?

   Yes No I do not know or I do not remember

6. Is there a good physical reason not mentioned here why you should not follow an activity program even if you wanted to?

   Yes No I do not know or I do not remember

7. Are you over age 65 and not accustomed to vigorous exercise?

   Yes No I do not know or I do not remember

If a participant answers yes to any question, vigorous exercise, or exercise testing should be postponed until medical clearance is obtained. "I do not know" answers should be researched further to determine testing suitability.
