# Supplementary material for: Internet-Based Exercise Therapy Using Algorithms for Conservative Treatment of Anterior Knee Pain: A Pragmatic Randomized Controlled Trial
Source: JMIR Rehabil Assist Technol. 2016 Dec 14;3(2):e12. doi: 10.2196/rehab.5148 (PMC5454560; doi:10.2196/rehab.5148)
Supplement: Supplementary file 2 [file rehab_v3i2e12_app2.pdf]

Multimedia Appendix 2. Knee Exercise Eligibility Score (KEES)

1. *Do you have knee pain?*
   (Correct answer=Yes)

2. *Have you ever been told you should only do physical activity that has been recommended by a Doctor?*
   (Correct answer=No)

3. *Do you ever have pain in your chest when exercising?*
   (Correct answer=No)

4. *When exercising do you ever get dizzy, lose your balance or fall?*
   (Correct answer=No)

5. *Do you have any other reason why you should not exercise?*
   (Correct answer=No)

6. *Does you knee currently have painful swelling?*
   (Correct answer=No)

7. *Are you able to walk without assistance (cane, walker, etc.)?*
   (Correct answer=Yes)

8. *Do you have clicking, locking, popping in your knee OR pain that awakens you from sleep?*
   (Correct answer=No)

9. *Have you had knee surgery in the past year or has your doctor told you that you need a knee surgery?*
   (Correct answer=No)

10. “Do you have a workers comp claim or pending litigation?

(Correct answer = No)
