# Supplementary material for: Internet-Based Exercise Therapy Using Algorithms for Conservative Treatment of Anterior Knee Pain: A Pragmatic Randomized Controlled Trial
Source: JMIR Rehabil Assist Technol. 2016 Dec 14;3(2):e12. doi: 10.2196/rehab.5148 (PMC5454560; doi:10.2196/rehab.5148)
Supplement: Supplementary file 3 [file rehab_v3i2e12_app3.pdf]

Multimedia Appendix 2. Informed Consent Document

APPROVED BY RCRC IRB: 13 AUGUST 2012 EXPIRES: 13

JUNE 2013

AN AGREEMENT TO BE IN A RESEARCH STUDY
INFORMED CONSENT DOCUMENT

**Sponsor:**	Jonathan P. Garino, MD
**City and State:**	*Philadelphia, Pennsylvania*
**Protocol Number and Title:**	413; *“Conventional home exercise programs versus electronic home exercise versus artificial intelligence “virtual therapy” for anterior knee pain: a prospective randomized comparative trial”*
**Study Doctor:**	Jonathan P. Garino, MD
**Address of Study Site(s):**	835 Stoke Road Villanova, PA 19085
**24-Hour Telephone Number:**	(215) 516-9660, (214) 471-3984

INTRODUCTION

You are being asked to take part in a medical research study. Before you decide to take part in this study, you should read this document. This document, called an informed consent document, explains the study. Please ask as many questions as needed so that you can decide if you want to be in the study.

To be in this research study, you cannot already be in another medical research study.

You must be honest and complete in providing your medical history. Giving false, incomplete, or misleading information about your medical history could have very serious health consequences.

The study doctor has a financial interest of the outcome of the study.

PURPOSE OF THE STUDY

The purpose of this study is to compare a new method of delivering exercise therapy, called Simple Therapy, to what exists today. You will be randomly assigned, (like a flip of coin), to one of three methods.

- conventional non-supervised home exercise program with online handouts
- non-supervised exercise program with 6 exercise videos
- virtual home exercise program that changes the exercises based on user input with Simple Therapy The study

is single-blinded, meaning that only you, the user, will know which method you are using.

WHAT WILL HAPPEN DURING THE STUDY

You will first have to answer a questionnaire to help decide if you can be in the study. This is called “screening.”

Screening does not guarantee entry into the study. Entry into the study will depend upon the results of the answers you gave to the questions and study specific guidelines. Even if you pass the screening tests, there is a chance that you will not be invited to take part in the study. There may be other reasons why you cannot take part in the study.

In order to participate in the study you must meet the following criteria:

- Have knee pain

APPROVED BY RCRC IRB: 13 AUGUST 2012

EXPIRES: 13 JUNE 2013

- Be 18 years to 64 years old
- Have passed the PAR-Q and have been cleared by a medical professional for exercise
- If you don't have access to a medical professional, the study team can provide medical clearance by way of a phone conversation, for the purpose of the trial.
- Have access to the internet
- Have agreed to the Terms of Service and Informed Consent

LENGTH OF THE STUDY AND NUMBER OF PARTICIPANTS EXPECTED TO TAKE PART IN THE STUDY

966 participants, over the age of 18 and less than 65 are expected to participate in this study. You will be in this study for up to *90* days; this begins with your screening visit, and continues until your last study survey. During the study you will have:

- to perform 3 sessions of 6 exercises per session per week for a total of 6 weeks.
- to complete an online questionnaire about your condition before the first exercise and at week 3, week 6 and week 12.

For users assigned to Simple Therapy, at the end of each exercise session you will provide input on the difficulty of the exercises via a website. Based on this feedback, another video based exercise regimen will be provided for the next scheduled session. This process will continue until the full 6-week session is completed.

Each exercise session will be approximately 12-18 minutes in duration.

SIDE EFFECTS AND OTHER RISKS

One of the reasons for this study is also to learn more about the possible side effects of performing non supervised exercise and exercise with Simple Therapy over the Internet. It is important that you tell the study staff about all side effects that you have.

The most common side effects of the study include:

- Increased pain in your joints
- Delay of diagnosis of any medical condition

If you are not honest about your side effects, it may not be safe for you to stay in the study.

UNFORESEEABLE RISKS:

Participating in this study may involve risks to you that are presently unforeseen and unknown.

POSSIBLE BENEFITS OF THE STUDY

There is no promise that your condition will get better; it can stay the same or even get worse.
Information learned from the study may help other people with the same knee problem in the future.

PAYMENT FOR BEING IN THE STUDY

You will be contacted by the study team for interval survey followup, and you may be provided an electronic gift card at interval(s) determined by the study team.

You will receive $50 for the completion of the follow-up survey. If you do not complete the survey, you will not be paid. You will receive payment about 4-6 weeks after the survey is completed.

ADDITIONAL COSTS

There are no additional costs associated with this study.

APPROVED BY RCRC IRB: 13 AUGUST 2012

EXPIRES: 13 JUNE 2013

ALTERNATIVES TO PARTICIPATION

There are other alternatives to relieve knee pain that are available if you decide not to be in the study. These alternatives include seeking treatment by a physician, a physical therapist or other medical professionals. You and your personal doctor can decide what treatment is best for you.

RELEASE OF MEDICAL RECORDS AND PRIVACY

Records of you being in this study will be kept private. There may be times when the study staff will not be able to guarantee privacy, such as when your study medical records are requested by a court of law or when shared with a firm in another country that does not have privacy regulations in place. **If information from this study is published or presented at scientific meetings, your name and other personal information will not be used.** The following people will have access to your study records:

- Study Doctor
  - Sponsor Company or Research Institution
  - The United States Food and Drug Administration (FDA)
  - The Department of Health and Human Services (DHHS)
  - Other State or Federal Regulatory Agencies
  - RCRC Independent Review Board (IRB)

RCRC and accrediting agencies may inspect and copy your records, which may have your name on them. Therefore, your total privacy cannot be guaranteed.

A description of this clinical trial will be available on *http://www.ClinicalTrials.gov*, as required by U.S. Law. This Web site will not include information that can identify you. At most, the Web site will include a summary of the results. You can search this Web site at any time.

IN CASE OF AN INJURY RELATED TO THIS RESEARCH STUDY

Compensation for any unforeseen injury is not offered, but the study staff can provide guidance to seek professional medical assistance if needed. It is important that you tell your study doctor, if you feel that you have been injured because of taking part in this study. You can tell the study doctor in person or call him/her at the number listed on the first page of this consent document.

Please be aware that some insurance plans may not pay for research-related injuries. You should contact your insurance company for more information.

LEGAL RIGHTS

You do not lose any legal rights by signing this consent document. The above statement, "In Case of an Injury Related to This Research Study," does not stop you from getting legal help in case of negligence.

NEW FINDINGS

During the study, you will be told of any important new findings about the study. You can then decide if you still want to be in the study.

APPROVED BY RCRC IRB: 13 AUGUST 2012

EXPIRES: 13 JUNE 2013

WHOM TO CONTACT

You may contact the Study Doctor or study staff at the phone number listed on the first page of this consent document:

- for answers to questions, concerns, or complaints about this research study
- to report a research related injury, or
- for information about study procedures.

If you need medical attention please go to the nearest emergency room.

You may contact RCRC if you:

- would like to speak with someone not related to the research,
  - have questions, concerns, or complaints regarding the research study, or
  - have questions about your rights and welfare as a research participant.

Chairman, RCRC Independent Review
Board 2111 West Braker Lane, Suite 400
Austin, TX 78758
Or you may email rcrc@rcrcirb.com
Or you can call: 855-300-0815 between 8:00 AM and 5:00 PM Central Time

If you would like additional information, you may visit RCRC's website at www.rcrcirb.com.

RCRC has approved this study and this informed consent document. RCRC is a group of scientific and non-scientific people who review, and approve or disapprove research involving people by following the federal regulations. This group is also required by the federal regulations to do periodic review of ongoing research studies.

LEAVING THE STUDY

Taking part in this study is your choice. You may choose either to take part or not to take part in the study. You have the right to leave this study at any time. If you do not want to be in the study, there will be no penalty to you, and you will not lose any benefits to which you are otherwise entitled.

If you wish to leave this study, please call the Study Doctor or study staff at the telephone number listed on the first page of this consent document to schedule study exit procedures.

If you withdraw from the study, the study doctor will request that the final survey questionnaire be filled out. The study doctor will inform you whether the study doctor intends to either: (1) retain and analyze already collected data relating to you up to the time of your withdrawal; or (2) honor your request that the study doctor destroy the your data or that the study doctor exclude your data from any analysis.

Your part in this study may be stopped at any time without you being asked. The following people can stop your participation and/or the study itself:

- The Study Doctor
- RCRC Independent Review Board
- Other State and Federal Regulatory Agencies
  - The Sponsor Company

If you do not follow the study procedures you may be taken out of the study.

APPROVED BY RCRC IRB: 13

AUGUST 2012 EXPIRES: 13 JUNE

2013

<u>AGREEMENT TO BE IN THE STUDY</u>
This consent document contains important information to help you decide if you want to be in this study. If you have any questions that are not answered in this consent document, please ask the person explaining this document or one of the study staff. You will have ample opportunity to email any questions you have about the study. If required, you may place a phone call to the study doctor and all of your questions will
be addressed. Once all your questions and concerns have been addressed, we will obtain an electronic signature.

By consenting to participate you agree that you have been given a copy of all pages of this consent document. You have had an opportunity to ask questions and received satisfactory answers to all your questions about this study. You understand that you are free to leave the study at any time without having
to give a reason and without affecting your medical care. You understand that your study-related medical records may be reviewed by the company sponsoring the study and by government authorities.

IF YOU DO NOT AGREE WITH THE STATEMENT ABOVE, YOU SHOULD NOT SIGN THIS INFORMED CONSENT DOCUMENT.

Printed Name of Adult Participant

Signature of Adult Participant Date
DO NOT SIGN AFTER 13 JUNE 2013

Printed Name of Person Explaining Informed Consent Document

Signature of Person Explaining Informed Consent Document Date
