# Supplementary material for: Internet-Based Exercise Therapy Using Algorithms for Conservative Treatment of Anterior Knee Pain: A Pragmatic Randomized Controlled Trial
Source: JMIR Rehabil Assist Technol. 2016 Dec 14;3(2):e12. doi: 10.2196/rehab.5148 (PMC5454560; doi:10.2196/rehab.5148)
Supplement: Supplementary file 4 [file rehab_v3i2e12_app4.pdf]

Multimedia Appendix 4

<u>Initial Questionnaire (Before first exercise session) - "Tell us About Yourself"</u>

1. Imagine that before you had pain you were at 100%. What % is your knee right now? (Slider from 0-100- *General Assessment of Function*.)

2. What sports and activities could you do before the pain? (Slider 0-10 *UCLA Activity Score*)

3. What sports & activities can you do right now? (Slider 0-10 *UCLA Activity Score*)

4. If 10 is the worst pain you can imagine, rate your pain now from 1-10. (Slider 0-10 with "Face figures"- *Visual Analog Scale*)

5. How many visits to a physical therapist have you ever had?

    0

    1-3

    4-8

    8-15

    Greater than 15

6. Has your knee pain ever caused any of the following? (Check all that apply):

    Visit a Doctor

    Visit a Physical Therapist

    Receive an injection

    Be in the hospital

6 weeks Questionnaire - "Now that you are done with your exercises, tell us how it went."

1. Imagine that before you had pain you were at 100%. What % is your knee right now? (Slider from 0-100- *General Assessment of Function*.)

2. What sports & activities can you do right now? (Slider 0-10 *UCLA Activity Score*)

3. If 10 is the worst pain you can imagine, rate your pain now from 1-10. (Slider 0-10 with "Face figures"- *Visual Analog Scale*)

4. Are you feeling better than when you started? Yes/No

Multimedia Appendix 4

5. Do you like this better than going to a physical therapist? Yes/No

6. What would you pay for 6 weeks of unlimited website access? Open text box, numbers only

7. During the trial, did the knee pain ever cause you to: (Check all that apply):

Visit a Doctor

Visit a Physical Therapist

Receive an injection

Be in the hospital

8. Did you like your exercise program? Yes/No

9. Would you recommend your exercise regimen to friends or family?

10. What was the biggest reason you tried the website?

Cost

Ease of use

Ease of access

Effectiveness

Other: (free text)
